# Molecular characterization of multidrug-resistant *E. coli* recovered from diarrheagenic children under 5 years from Mukuru Informal Settlement, Nairobi, Kenya, based on whole-genome sequencing analysis

**DOI:** 10.1128/spectrum.01420-24

**Published:** 2025-05-15

**Authors:** Susan Kiiru, Purity Kasiano, John Maina, John Njeru Mwaniki, Edinah Songoro, Samuel Kariuki

**Affiliations:** 1 Center for Microbiology Research, Kenya Medical Research Institute 118982, Nairobi, Kenya; 2 Jomo Kenyatta University of Agriculture and Technology, JKUAT 118985, Nairobi, Kenya; 3 Drugs for Neglected Diseases initiative, Eastern Africa, Nairobi, Kenya; Rush University Medical Center, Chicago, Illinois, USA

**Keywords:** multidrug-resistant, *E. coli*, diarrhea, children under five years, informal settlement

## Abstract

High genomic plasticity within *Escherichia coli* enables it to acquire and accumulate genetic material through horizontal gene transfer. In this study, we sought to investigate the virulence genes, phylogroups, antibiotic resistance genes, plasmid replicons, multilocus sequence types (MLST), and core genome MLST of multidrug-resistant *E. coli* recovered from diarrheagenic children under 5 years from Mukuru Informal Settlement in Nairobi, Kenya. A total of 39 multidrug-resistant (MDR) strains had their DNA extracted, and whole-genome sequencing was done using the Illumina HiSeq 2000 platform. Twenty-six *E. coli* assemblies were analyzed using web-based bioinformatics tools available at the Centre for Genomic Epidemiology and EnteroBase. The isolates were categorized into four main phylogroups, where 10/26 (38.5%) belonged to the B2 phylogroup, 4/26 (15.4%) belonged to D, 3/26 (11.5%) belonged to A, 1/26 (3.8%) belonged to B1, while 8/26 (30.8%) were not determined. FimH30 was predominantly found in the most frequent phylogroup B2 and sequence type (ST) 131. The most common beta-lactam resistance genes were *bla*_TEM-1B_ and *bla*_CTXM 15_, followed by three fluoroquinolone resistance genes [*qnrS1* 6/26 (23.1%), *qnrB4* 2/26 (7.7%), and *aac(6′)-Ib-cr*, 8/26 (30.8%)]. Of 26 isolates, 15 had at least one amino acid substitution in the housekeeping genes *gyrA* (*p.S83L*), *gyrA* (*p.D87N*), *parC* (*p.S80I*), *parC* (*p.E84V*), *parC* (*p.S57T*), and *parE* (*p.I529L*), associated with resistance to fluoroquinolones. A total of 40 diverse virulence genes were detected among the isolates. Thirteen different STs were isolated from the *E. coli* genomes, which included ST 131, ST 3036, ST 38, ST 10, ST 12569, ST 15271, ST 2076, ST 311, ST 3572, ST 394, ST 453, ST 46, and ST 1722. Only two isolates (2/26, 7.7%) from the Municipal City Council clinic were genetically related. Additionally, the most abundant plasmid replicon identified belonged to the IncF family, IncFII(pRSB107), in particular, followed by the Col family. The study highlighted the first *E. coli* ST46 to harbor the *bla*_NDM5_ gene encoded in Col(BS512), IncFII(pRSB107), and IncFIB(AP001918) plasmid replicons in Kenya. We further demonstrated the diversity of MDR *E. coli* associated with diarrhea in an endemic setting in Kenya.

**IMPORTANCE:**

This study investigated the molecular characterization of multidrug-resistant *Escherichia coli* isolated from diarrheagenic children under 5 years of age in Mukuru Informal Settlement in Nairobi, Kenya. This is an important addition to the genomic analysis data of multi-drug resistant diarrheal *Escherichia coli* in Kenya. The use of whole-genome sequencing to identify and characterize these isolates is valuable and provides valuable insights into the molecular epidemiology of *E. coli* in the region.

## INTRODUCTION

Diarrheagenic *Escherichia coli* is implicated in 80% of diarrheal illnesses in humans, causing 70,000 deaths in children under 5 years of age in Africa (1). Several clinical cases involving children below 5 years of age suffering from diarrhea are due to multidrug-resistant (MDR) diarrheagenic *E. coli* (DEC) (2–4). From MDR diarrheagenic *E. coli*, the extended-spectrum ß-lactamase (ESBL) and carbapenemase-producing DEC pathotypes are among the emerging pathogens globally (5). Pathogenic and non-pathogenic *E. coli* are classified into A, B1, B2, C, D, E, and F phylogenetic groups based on the possession of *chuA*, *yjaA*, *tspE4.C2,* and *arpA* genes. The human pathogenic *E. coli* are commonly classified into the B2 or D phylogroups, whereas commensal and less virulent strains are classified into the A or B1 group (6). Increasing antimicrobial resistance among pathogenic *E. coli* strains and the horizontal genetic transfer mechanism allow the exchange of resistance genes within and between phylogroups, which may increase the emergence of resistant strains belonging to commensal strains (6). Moreover, commensal bacteria can harbor and exchange resistance and virulence genes through horizontal gene transfer and provide a reservoir of resistance genes, which may be transferred between bacterial species, including pathogens (7).

According to the World Health Organization’s list of pathogens of critical priority for research and development of antibiotics, extended-spectrum β-lactamase (ESBL)-producing *E. coli* tops the list (8, 9). The ESBL *E. coli* have a wide β-lactam hydrolysis capability extended to even advanced antimicrobial agents, such as penicillins, 1st-, 2nd-, 3rd-, and 4th-generation cephalosporins, and monobactam, but not cephamycins (10). The mechanism of resistance toward β-lactam antibiotics, such as cefotaxime and ceftriaxone, has predominantly been attributed to the production and spread of β-lactamase proteins that can hydrolyze these agents (9, 11). To date, *bla*_TEM_, *bla*_CTX-M_, *bla*_SHV_, and *bla*_OXA_ are the most reported β-lactamases in enteric pathogens reported in Kenya (12, 13). Transmission and acquisition of ESBLs, among other antimicrobial resistance genes, have been mainly facilitated by horizontal transfer through plasmid-borne integron.

In most Kenyan outpatient hospitals, fluoroquinolones are among the commonly used treatment options for diarrheal infections (14). Fluoroquinolone resistance mediated by bacterial species is due to point mutations in the quinolone resistance-determining region, *gryA*, and *parC* gene. The co-existence of the quinolone-resistance-determining region and plasmid-mediated quinolone-resistance, such as *qnrS* and *qnrB*, aggravates resistance to this class of antimicrobial agents (15). Plasmids can mobilize and transmit multiple antimicrobial resistance genes with and across bacterial species. The *aac(6’)-lb-cr* gene, which encodes aminoglycoside acetyltransferase, is also capable of causing combined resistance to fluoroquinolones and aminoglycosides (16). Fluoroquinolones, such as ciprofloxacin and norfloxacin, may also be resistant through the efflux pump mechanism mediated by the *qepA* gene. Similarly, transposable elements encoding New Delhi metallo-β-lactamase (*NDM*) genes have been identified as the cause of the rapid spread of carbapenem resistance in *E. coli* (17).

Different virulence factors contribute to bacterial pathogenic potential, such as adhesive factors used by bacteria for primary colonization, iron acquisition system, and production of various toxic substances to invade the cells and overcome the host immune response (18). For *E. coli*, four unique virulence genes are used to distinguish them into pathotypes, namely, colonization (*agg3A*, *fimH*, *csgA*, and *tir*), fitness (*kpsMT II*, *fyuA*, *iutA*, and *iucB*), toxins (*stx1*, *stx2*, *LT I*, and *hlyE*), and effectors (*ipaH*, *virB*, *espH*, and *espA*) (18). Additionally, the transmission of *E. coli*, directly or indirectly, through a common source can be inferred by closely related genomes in different individuals. The closely related genomes in different people are said to be clonal strain sharing (19). Sequence types (ST) using the Achtman seven-allele system and core genome multilocus sequence typing (cgMLST) are used to assess clonality (20). cgMLST has high accuracy and can divide strains with minor sequence differences into different cgMLST sequence types (cgSTs), providing a powerful typing approach for molecular epidemiologic investigations (21). In Kenya, previous studies have looked into the prevalence and molecular basis of antimicrobial resistance (AMR) in clinical *E. coli* using low-resolution phenotyping methods that do not shed light on the genotypic diversity of the strains and/or causes of AMRs (2, 12, 13, 22). Limited data on genomic characterization of MDR diarrheagenic clinical *E. coli* are available. In this study, we sought to investigate the virulence genes, phylogroups, antibiotic resistance genes (ARGs), plasmid replicons, multilocus sequence types (MLST), and cgMLST of multidrug-resistant *E. coli* recovered from children under 5 years with diarrhea from Mukuru Informal Settlement in Nairobi, Kenya.

## MATERIALS AND METHODS

### Sample collection and microbiological processes

Children with diarrhea (≤5 years) were recruited from outpatient clinics of the Municipal City Council, Mukuru kwa Reuben, Mary Mother Mission, and Mama Lucy Kibaki Hospital, Nairobi using a cross-sectional design. A healthcare worker at each of the clinics identified patients presenting mainly with abdominal pain and intense and frequent urge for bowel movement, accompanied by other symptoms, including vomiting, fever, dehydration, weight loss, and loose stool. Participants who had taken antibiotics within the previous 7 days to treat other illnesses were excluded from the study, as confirmed by the clinician and the guardian/parent of the child. The parents/guardians of the participants meeting the inclusion criteria were interviewed to obtain the history of their illness, and then asked to provide a stool sample or a rectal swab. However, we did not recruit inpatient children and those who were being given parenteral antibiotics.

A total of 219 stool samples were collected from the children between July 2021 and November 2021. Stool culture was done on MacConkey and *Salmonella Shigella* agars, while the recovered bacteria were identified using VITEK 2GNID and PCR, where the protocol used for the PCR is well documented in the previous work (23). Antibiotic susceptibility testing (AST) for 157 non-duplicate representative isolates was done using VITEK 2AST-GN83, as previously described (23). Isolates exhibiting resistance to three or more classes or subclasses of antibiotics were scored as MDR as defined by the European Center for Disease Control (24). A total of 39 MDR strains were identified, that is, 27 *E. coli*, two *Enterobacter* spp., three *Klebsiella* spp., one *Morganella morganii*, one *Providencia alcalifaciens*, one *Proteus mirabilis*, one *Salmonella* spp., two *Shigella* spp., and one *Kluyvera cryocrescens*, and had their whole genome sequenced.

### Whole-genome sequencing

The DNA of 39 MDR isolates was extracted using GenElute Bacterial Genomic DNA Kit (Sigma Aldrich) following the manufacturer’s instructions (25). DNA quality and quantification were determined on the NanoDrop One (Thermo Fisher Scientific, Waltham, MA, USA) at the Kenya Medical Research Institute, Centre for Microbiology Research, Nairobi, Kenya. Pure genomic DNA was transported under the material transfer guidelines from KEMRI to Ohio State University. The library size and concentration were determined using the 4150 TapeStation System (Agilent, MA, USA). Limited-cycle PCR was subsequently employed to amplify the tagged DNA and introduce sequencing indexes. To facilitate a limit of detection assessment for each sample, we incorporated 25 pg of the External RNA Controls Consortium (ERCC) RNA Spike-In Mix (Life Technologies, Carlsbad, CA, USA) into each sample prior to library preparation. The prepared libraries were loaded onto a reagent cartridge and subjected to clustering on the NextSeq 2000 System. Subsequently, a paired-end sequencing run with 2 × 150 bp reads was executed using the NextSeq 2000 platform. The base calls generated by the NextSeq 2000 System were then transformed into FASTQ files. To manage the NGS data and identify potential quality issues before downstream bioinformatic analysis, we employed FastQC v.11.7 (Babraham Bioinformatics). We conducted an initial assessment of quality-related metrics, including cluster density, *q*-scores, and the percentage of passed reads as provided by the sequencer, using FastQC.

### Genome assembly and species identification

Raw sequence reads were *de novo* assembled using the Global Health Research Unit for genomic surveillance of the antimicrobial resistance pipeline (https://gitlab.com/cgps/ghru/pipelines/assembly). Briefly, read trimming and removal of adapter were done using Trimmomatic (0.38) (26), correction of reads using Lighter (1.1.1) (27), merging the reads using Flash (1.2.11) (28), and assembly using SPAdes (3.12.0) (29). Quality control was done using FastQC (0.11.8) (30) and MultiQC (1.7) (31). Contamination was checked using Confindr (0.7.2) (32). Species identification was carried out by Bactinspector (0.1.3) (33), and the concordance of bacteria identity between Vitek and whole-genome sequencing (WGS) identities was compared. The assembly metrics were also assessed using QUAST version 5.0.2 (34).

### Prediction of antimicrobial resistant genes, virulence genes, and phylogroup determination

The assembled genomes were investigated for ARGs using ResFinder 4.4.2 using the default threshold of minimum accepted alignment of 60% and minimum accepted identity of 90% and housed in the Centre for Genomics Epidemiology of the Technical University of Denmark (https://cge.food.dtu.dk/services/ResFinder/). The plot of ARG stratified by phenotypic ASTs was represented in a heatmap plotted using GraphPad Prism (https://www.graphpad.com/features). Concordance between phenotypic and genotypic AMR was also determined, where the organisms were checked for detection of ARG in phenotypically susceptible isolates and the presence of ARG in phenotypically resistant isolates. For the whole-genome sequencing data, a resistant score was given when one or several ARGs or chromosomal mutations were revealed in Resfinder and confirmed to be the mechanism of resistance to the antimicrobial, and a susceptible score was assigned when there were no ARGs. The virulence genes were also investigated using virulenceFinder version 2.0 (https://cge.food.dtu.dk/services/VirulenceFinder/), with *E. coli* species and 90% threshold % identity and 65% minimum length, and the distribution of the virulence genes per phylogroup was represented using bar plots that were visualized using the *ggplot* function from the Tidyverse package (v1.3.1) in R. The *E. coli* phylogroups were determined using the Enterobase *E. coli/Shigella* database under experiment data phylogroups that employed Clermont typing (https://enterobase.warwick.ac.uk/species/ecoli/search_strains), as well as FimH typing, which was ascertained using FimTyper version 1.0 under https://cge.food.dtu.dk/services/FimTyper/.

### MLST, cgMLST, and plasmid determination

The MLST of *E. coli* were determined using the Enterobase *E. coli/Shigella* database under experiment data Achtman seven-gene MLST. The results were confirmed using MLST version 2.0.9 under https://cge.food.dtu.dk/services/MLST/. Moreover, the transmission of *E. coli* within and between the four recruitment sites, directly or indirectly, inferred by isolates with closely related genomes in different individuals was determined using core genome MLSTs. The cgMLSTs were determined using the Enterobase *E. coli/Shigella* database under experiment data cgMLST V1 + HierCC V1, Algorithm MSTree V2 based on pairwise differences between genomes at cgMLST alleles, and a grape minimum spanning tree was constructed. The branch lengths of the grape tree reflect the number of allele differences between the allelic profiles of the isolates in the connected nodes. On the contrary, plasmid replicons were identified using PlasmidFinder version 2.0.1 (https://cge.food.dtu.dk/services/PlasmidFinder/) with Enterobacteriales database, 95% minimum % identity, and 60% minimum % coverage. PLASME was also used to identify plasmid contigs from the short read assemblies using transformer (35).

## RESULTS

### Species identification and phenotypic antimicrobial susceptibility testing

Of the 39 MDR bacteria isolates sequenced, one isolate failed at the quality control step and could not be assembled and, hence, was excluded from the study. Of the 38 assembled genomes, 26 were *E. coli*; two were *Enterobacter cloacae*; two were *Enterobacter kobei*; one was *Enterobacter hormaechei*; one was *Enterobacter asburiae*; one was *Enterobacter bugandesis*; two were *Klebsiella pneumoniae*; two were *M. morganii*; and one was *P. alcalifaciens*. Six out of the 27 (22.2%) *E. coli* identified by the Vitek2 system were reclassified by Bactinspector as two *E. kobei*, one *K. pneumoniae*, one *P. alcalifaciens*, one *E. asburiae*, and one *E. cloacae*. The Vitek 2 *M. morganii*, *Salmonella*, *Klebsiella oxytoca*, *P. mirabilis*, and *K. cryocrescens* were identified as *E. coli* by WGS. Moreover, the Vitek 2 *E. cloacae* was identified as *K. pneumoniae*, and *K. pneumoniae* was identified as *E. hormaechei*. The manuscript will only focus on the genomic characterization of *E. coli* from the correctly identified species.

All isolates were resistant to ampicillin, cefazolin, and sulfamethoxazole-trimethoprim at (24/26) 96, (24/26) 96, and (22/26) 85%, respectively. All isolates were susceptible to amikacin, except for only one isolate. Moreover, the isolates were susceptible to meropenem, except for three isolates, where two were fully resistant, and one was intermediate. The resistance toward beta-lactams (cefuroxime, cefuroxime axetil, ceftriaxone, cefotaxime, and ceftazidime) ranged from 30 to 76%. Among the β-lactamase inhibitors (amoxicillin clavulanic acid, ampicillin-sulbactam, and tazobactam piperacillin) tested, ampicillin sulbactam was the least effective at 73%. The isolates were resistant to ciprofloxacin and aztreonam at (14/26) 53%, while nitrofurantoin, gentamicin, cefoxitin, and cefepime were at 23 to 34%.

### FimH typing and phylogroups of *E. coli*

The sequenced *E. coli* genomes were classified into four main phylogroups, where 10/26 (38.5%) belonged to the B2 phylogroup; 4/26 (15.4%) belonged to D; 3/26 (11.5%) belonged to A; 1/26 (3.8%) belonged to B1; and 8/26 (30.8%) were not determined. Moreover, 10 unique FimH types were identified. FimH30 was the most prevalent at 7/26 (26.9%) and predominantly found in phylogroup B2 and ST 131. Other FimH included FimH54, 5/26 (19.2%), FimH41, 2/26 (7.7%), each of FimH153, FimH31, FimH47, FimH25, FimH30, FimH5, and six unknown.

### Virulence genes associated with *E. coli* genomes

**Fig 1:**
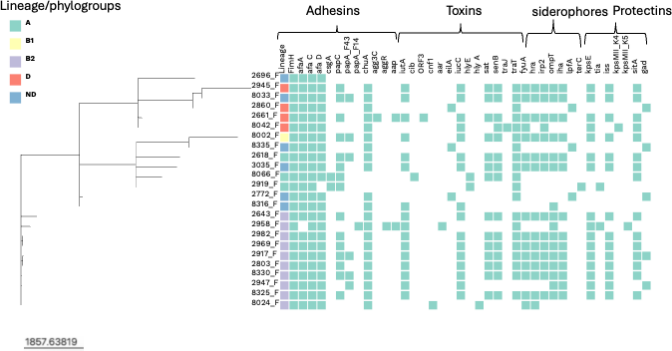
A heatmap showing the presence and absence of adhesins, toxins, siderophores, and protectins virulence genes within each phylogroup. White represents absence, while Bermuda green represents gene presence. The tree scale shows cgMLST allelic differences per isolate.

A total of 40 diverse virulence genes were detected. The common virulence genes involved in colonization, fitness, toxins, and effectors were detected in these isolates. The outer membrane hemin receptor (*chuA*), siderophores (*fyuA, irp, yfcV*), hemolysin (*hlyA and hlyE*), aerobactin (*iuc*), adhesin (*afaC, afaD*), polysialic acid transport protein (*kpsM11_K4* and *kpsM11_K5*), pyelonephritis-associated pili (*papC*), SPATE genes (*sat*), and plasmid-encoded *Shigella* enterotoxin *senB* were more abundant, Fig. 1. Out of 40 virulence genes, 10 (*cib*, *orf3*, *eilA*, *hlyE*, *ipfA*, *terC*, *agg3C*, *kpsmII_K4*, *papA_F14*, and *tra J*) were not found in phylogroup B2 but were present in phylogroups A, D, B1, and unknown. Virulence genes like *orf3*, *eilA*, *agg3C*, *kpsM11_K4*, *papA_F43*, and *traJ* were only present in phylogroup D, while *cnf1*, *hlyA*, and *kpsMII_K5* were only present in phylogroup B2 and *cib* and *hlyE* in phylogroup A.

### MLST and cgMLST

**Fig 2:**
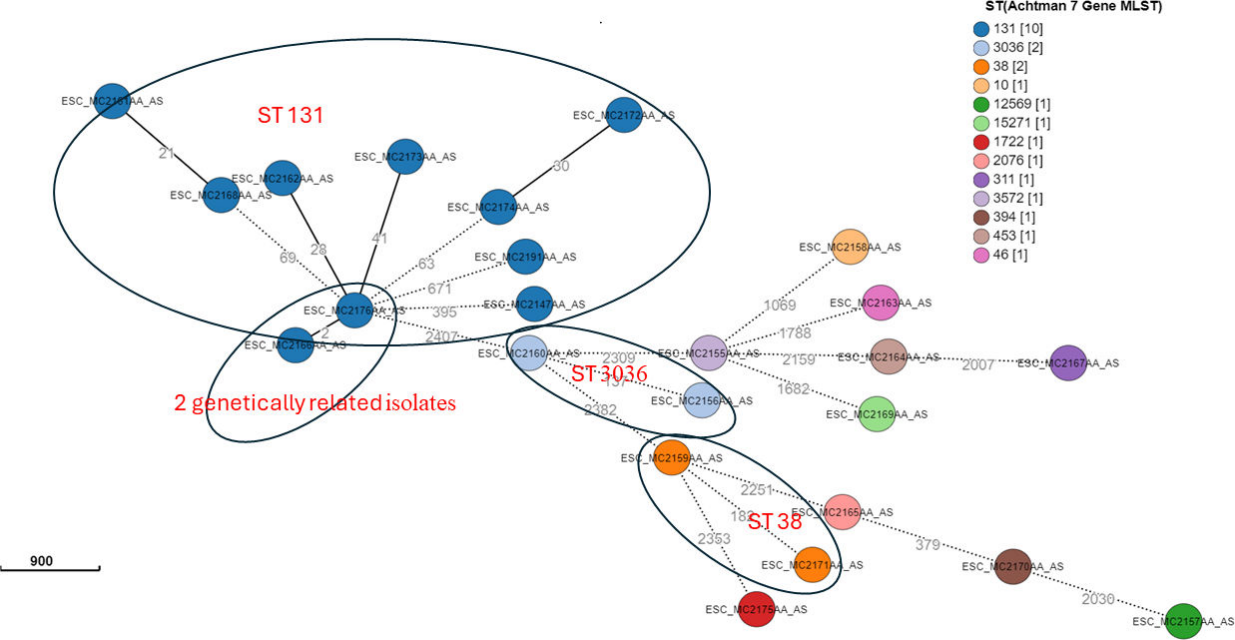
A minimum spanning tree of genetic relatedness of 26 *E. coli* based on cgMLST allelic profiles. The allelic differences (AD) numbers are indicated on the lines connecting the various cgMLSTs. The circle color represents STs according to the Warwick scheme (http://enterobase.warwick.ac.uk), and the strain IDs are indicated in the circles. Isolates in the same ST are circled and labeled.

Overall, 13 different STs were isolated from the *E. coli* genomes, which included ST 131 (12/26, 46.2%), ST 3036 (2/26, 7.7%), ST 38 (2/26, 7.7%), and one of each ST 10, ST 12569, ST 15271, ST 2076, ST 311, ST 3572, ST 394, ST 453, ST 46, and ST 1722. These sequence types were distributed across the four recruitment sites, except for ST 131, which was not isolated from Mama Lucy Kibaki Hospital (MLKH), an inpatient hospital. The STs found in this inpatient hospital were ST 1722, ST 2076, and ST 3036. Additionally, phylogroup B2 had only ST 131, and phylogroup B1 only had ST 311. The genetic relatedness was established where isolates with very closely related genomes clustered together according to their cgMLST. The isolates clustered according to ST, where they were categorized into four clonal complexes represented by ST 131, ST 3036, ST 3572, and ST 38, Fig. 2. One isolate within ST 131 (8024_F) served as the predicted founder in the minimum-spanning grape tree. Of the 26 *E. coli* strains, only two isolates (2/26, 7.7%) from the Municipal City Council (MCC) clinic within the ST 131 cluster were genetically related since they had two allelic differences, as shown in Fig. 2.

### Antimicrobial resistance genes and plasmid replicon associated with *E. coli* isolates

**Fig 3:**
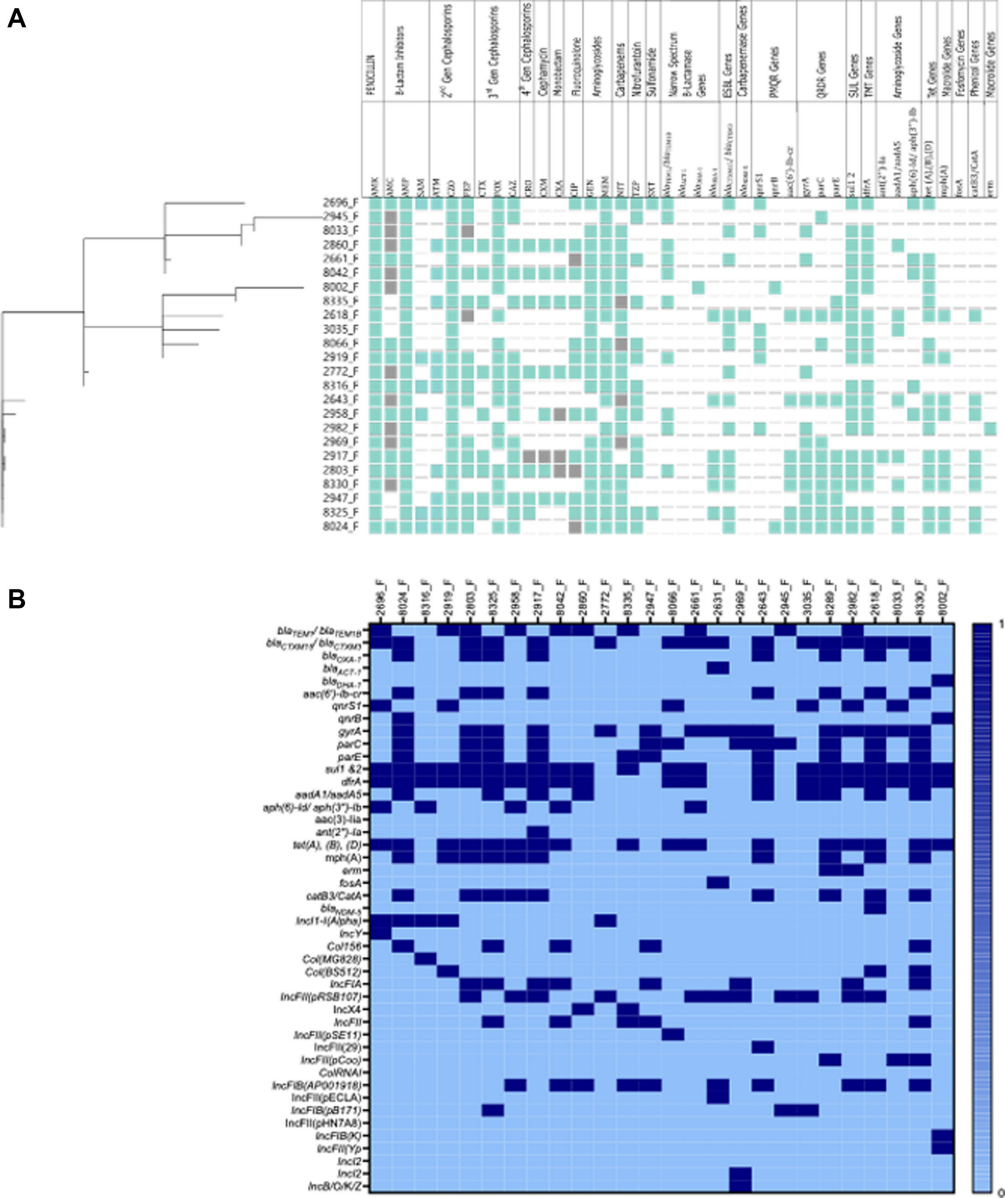
A heat map showing the phenotypic and genotypic profiles. The cladogram on the left indicates the clustering of *E. coli* isolates by cgMLST allelic profiles. For phenotypic profiles, susceptible isolates are indicated in Bermuda green, resistant isolates in white, and gray for intermediate isolates. The classes of antimicrobials are indicated on the top column header. For genotypic profiles, the presence of the gene is represented by Bermuda green and the absence white. The classes of the genes are indicated on the top column header. QRDR—quinolone resistance determining region; PMQR—plasmid-mediated quinolone-resistance; ESBL—expanded spectrum beta-lactamases; sul genes—sulfonamides resistance genes; TMT—trimethoprim resistance genes; and TET genes—tetracycline resistance genes.

In this study, beta-lactam resistance genes, which included *bla*_TEM1_, *bla*_TEM 1B_, *bla*_CTXM 15_, *bla*_CTXM 3_, bla_ACT-1_, *bla*_DHA-1_, and *bla*_OXA-1_, were detected, Fig. 3A. Three fluoroquinolone resistance genes [*qnrS1* 6/26 (23.1%), *qnrB4* 2/26 (7.7%), and *aac(6′)-Ib-cr,* 8/26 (30.8%)] were also detected. Moreover, *aadA1*, *aadA5*, *aph(3″)-Ib*, and *aph(6)-Id*, *ant (2″)-Ia*, *aac(3″)-Id* associated with aminoglycoside resistance were also detected. Other AMR genes that were detected were associated with trimethoprim resistance (*dfrA1*, *dfrA7*, *dfrA15*, *dfrA14*, and *dfrA17*), macrolide resistance (mphA, erm B), sulfonamide resistance (*sul1*, *sul2*), carbapenem resistance (*ndm-5*), tetracycline resistance [*tet(A)*, *tet(B)*, and *tet(D)*], and phenicol resistance (*catA1*, *catB3*). Of 26, 15 had at least one amino acid substitution in the housekeeping genes *gyrA* (*p.S83L*), *gyrA* (*p.D87N*), *parC* (*p.S80I*), *parC* (*p.E84V*), *parC* (*p.S57T*), and *parE* (*p.I529L*) associated with resistance to fluoroquinolones. A comparison of the isolates’ phenotypic and genotypic antimicrobial susceptibility results demonstrated a 77% concordance between phenotypically resistant isolates toward trimethoprim and sulfonamides and WGS resistant toward trimethoprim and sulfonamides. Additionally, there was 88% concordance between phenotypic resistance toward ciprofloxacin and WGS resistance. However, there was a 62% discordance between WGS resistance toward gentamicin and amikacin and 81% phenotypic susceptibility toward gentamicin and amikacin.

Additionally, the most abundant plasmid replicon identified from the study genomes belonged to the Inc F family, IncFII(pRSB107), in particular, followed by the Col family. Most isolates, 11/26, had two plasmid replicon types; 6/26 had one plasmid replicon; and 6/26 had five plasmid replicon types, Fig. 3B. Plasmid replicons, such as IncY, IncI2, Inc B/O/K/Z, IncFII(Pse11), IncFII(29), and Col(MG828), were found in only six individual isolates.

## DISCUSSION

*E. coli* genomic plasticity leads to the emergence of hybrid virulent and MDR strains, becoming significant etiologies of diarrhea outbreaks, particularly in children below 5 years of age in low- and middle-income countries (4). Robust detection of diarrheagenic *E. coli* and their genomic plasticity will provide public health information crucial for appropriately managing these infections. In this study, WGS revealed the misidentification of *E. coli* by the Vitek2 identification system. Twenty-one of 26 (80.1%) *E. coli* Vitek 2 ID concurred with the WGS Bactinspector species identification, while others were misidentified for *Enterobacter* spp*., Klebsiella* spp., and *P. alcalifaciens*. The findings are consistent with those from the study of Afolayan et al. (33), where *E. coli* was misidentified for *Enterobacter* spp. and *Klebsiella* spp. (33). The 5/26 (19.9%) identification discrepancies could be attributed to limitations in the Vitek 2 system. These results, therefore, highlight the need for a robust identification approach, such as WGS, to identify enteric bacteria associated with diarrhea for better treatment decisions.

Four main *E. coli* phylogroups, A, B1, B2, and D, were detected, with B2 being the most dominant at 38.5%, followed by D. These findings are in agreement with a study conducted in South Africa, where hospitalized children under 5 years were recruited, that showed 30.4% *E. coli* belonged to phylogroup B (36). These findings, however, contrast Richter and colleagues’ study conducted in Tanzania, where phylogroups A and B1 were the most dominant (37). These observed differences could be attributed to differences in the recruited participants. The current study recruited children under 5 years of age whose main symptoms were diarrhea, while Richter and colleagues’ study recruited children under the age of 5, and, other than a few who displayed diarrhea of an unknown source, were considered to be relatively healthy. Usually, *E .coli* strains classifying into B2 phylogroups are associated with extraintestinal infections. Therefore, our findings could imply that the intestinal microbiota is an important reservoir for bacteria that cause extraintestinal infections.

Moreover, ST 131 was the dominant sequence type among the *E. coli* genomes recovered from the outpatient clinics. This is unsurprising, as this ST is reported to be circulating globally (38, 39), with the recently conducted study in Kenya confirming the results (40). However, ST 131 was not isolated from the inpatient hospital, which could imply a change in patterns of the sequence type dominance in this setting. However, further studies need to be conducted to confirm our findings. The isolates also had high genetic diversity, where only two samples from MCC (8024_F and 8325_F) differed by only two cgMLST loci and had identical phenotypic and similar genotypic AMR, and plasmid profiles (8024_F has IncFII (pHN7A8 plasmid type), suggesting localized transmission of bacteria within the villages of this informal settlement. Since the two individuals came from the same village within this recruitment site, they are more likely to have contacted the same shared environment, increasing the chances of bacteria sharing.

Furthermore, *bla*_CTXM_ was the most abundant β-lactamase recovered, with *bla*_CTXM15_ being more dominant. This gene was detected with other ESBL determinants, *bla*_TEM_ and *bla*_OXA-1_ , in *E. coli* and *bla*_NDM5_. Consistent with a previous study conducted in a tertiary hospital in Dar es Salaam, *bla*_CTXM15_ was the most common (41). The detection of this gene in hospitals suggests that *bla*_CTX-M-15_-producing *E. coli* are a challenge to healthcare facilities in Africa. More urgently, there is a need to investigate the possible source and the transmission of *bla*_CTXM15_ in Nairobi, given the global concern about the spread of ESBL-producing *E. coli*.

Importantly, ST 131 *E. coli* presented resistance toward fluoroquinolones represented by single-amino acid substitutions at *gyrA (S83L*) and double-amino acid substitutions at *gyrA* (*S83L* and *D87N*) and *parC* (*S80I* and *E84V*). The double-amino acid substitutions in *gyrA* and *parC* resulted in high fluoroquinolone resistance compared to the plasmid-mediated quinolone resistance, *qnrS* and *qnrB*. As previously documented, the housekeeping gene mutations in *gyrA* and *parC* are typical of ST 131 *E. coli* (42, 43). Furthermore, there was 62% discordance in aminoglycosides (gentamicin and amikacin) tested in the study, where they were phenotypically susceptible but genotypically resistant. This discordance in a clinical setting where they heavily rely on empirical treatment could lead to inappropriate antimicrobial therapy and potentially worsen the diarrhea infection. Although the presence of resistance genes does not necessarily imply resistance, continued antimicrobial use could eventually lead to the expression of the resistance genes, which calls for the prudent use of antimicrobials.

Notably, only one isolate had New Delhi metallo-β-lactamase (NDM5) genes, which have been identified as the cause of the rapid spread of carbapenem resistance in *E. coli* (22). Alongside *bla*_NDM5_ was *bla*_CTX-M-15_, *gyrA* (*p.S83L*), *sul1*, *dfrA12*, *gyrA* (*p.D87N*), *mph(A)*, and *aadA2* genes, which have previously been reported to coexist (22). To the best of our knowledge, this is the first ST 46 *E. coli* to harbor the *bla*_NDM5_ gene in Kenya, as Musila and colleagues reported ST 167 and ST 648 harboring this gene (22). With increasing carbapenem use in Kenya because of the high levels of ESBL-producing Enterobacteriaceae, the detection of bla genesenes in *E. coli* poses a therapeutic threat to the treatment of diarrhea in Kenya, as carbapenems are considered the last-resort antibiotics. The most previously reported prevalent plasmids carrying the *bla*_NDM5_gene are IncFIA/B, IncFK, and IncX3 (44–46). In this study, however, this gene was harbored in Col(BS512), IncFII(pRSB107), and IncFIB(AP001918) plasmid replicons, and this calls for more studies to confirm this finding in Kenya.

Uniquely, the study reported more extraintestinal pathogenic *E. coli* (ExPEC) virulence genes than diarrheagenic *E. coli* virulence genes. The findings were also confirmed by a higher prevalence of phylogroup B2 associated with extraintestinal infections (47), agreeing with a study conducted in Nigeria with virulence genes significantly abundant in phylogroup B2 (33). The presence of ExPEC virulence genes in diarrheagenic children could imply their hybrid pathogenic potential. This phenomenon of high genomic plasticity within *E. coli* has been reported several times in most parts of the world (42). This contrasts with a study conducted in India that found diarrheagenic *E. coli* belonging to phylogroups B1 and A (6, 44), implying that different geographic locations have different patterns of infections, and these findings highlight that *E. coli* should not be regarded as non-pathogenic until its virulence genes have been investigated.

The study outcomes demonstrate the diversity of MDR *E. coli* associated with diarrhea in an endemic setting in Kenya. The study specifically showed unique genomic characteristics of *E. coli* in each of the four healthcare facilities, with two genetically related isolates being localized in the MCC clinic and the only *bla*_NDM5_ gene as the carbapenemase gene isolated from the MMM Clinic in Mukuru Informal Settlement. The high-risk clone ST 131 was found in MMM, MCC, and MR but not in MLKH, implying changes in dominance patterns. Since WGS is not frequently done, more studies investigating the genetic characteristics of MDR *E. coli* isolated from diarrheagenic children in Kenya are needed to confirm these findings and for a better basis of comparison.

### Limitations

Due to logistical constraints, only 157 samples were subjected to antimicrobial susceptibility testing, and there is a limited number of MDR strains sequenced. The study also looked at 26 *E. coli* genomes, which limited the results’ generalization.

### Conclusion

The recovered isolates were highly genetically diverse and spread across the four recruitment sites. The study also highlighted the first ST 46 *E. coli* to harbor the *bla _NDM5_* gene encoded in Col(BS512), IncFII(pRSB107), and IncFIB(AP001918) plasmid replicons in Kenya.

## Data Availability

The data that support the findings of this study are available from the corresponding author, but restrictions apply to the availability of these data, which were used under license for the current study and are not publicly available. Data are, however, available from the authors upon reasonable request and with permission of NACOSTI. The data sets generated and analyzed during the study have been deposited at DDBJ/ENA/GenBank under Bioproject PRJNA1065859 and genome accessions JAYWSW000000000-JAYWSX000000000, JAYXKY000000000-JAYXLS000000000. The versions described in this paper are versions JAYWSW01000000000-JAYWSX01000000000, JAYXKY01000000000, and JAYXLS01000000000 (https://www.ncbi.nlm.nih.gov/bioproject/PRJNA1065859).
